# Prevalence, Knowledge, and Practices of Hookah Smoking Among University Students, Florida, 2012

**DOI:** 10.5888/pcd11.140099

**Published:** 2014-12-04

**Authors:** Shams Rahman, Lissette Chang, Selamawit Hadgu, Abraham A. Salinas-Miranda, Jaime Corvin

**Affiliations:** Author Affiliations: Shams Rahman, Lissette Chang, Selamawit Hadgu, Abraham A. Salinas-Miranda, University of South Florida, Tampa, Florida.

## Abstract

**Introduction:**

Although hookah smoking is becoming a source of tobacco use among college students in the United States, little is known of the students’ knowledge, attitudes, and practices regarding hookah use. This cross-sectional study was aimed at determining the prevalence of hookah use and describing social and behavioral factors associated with hookah smoking among university students in a large urban university in Florida.

**Methods:**

A convenience sample of 478 undergraduate and graduate students was recruited. Lifetime use and current use was evaluated. Logistic regression modeling was used to assess the independent association between study covariates and hookah use.

**Results:**

Prevalence among students of having ever used hookah during their lifetime was 54.4%. Hookah use within the past 30 days was 16.3%. Hookah use was significantly associated with cigarette smoking (odds ratio [OR], 4.52; 95% confidence interval [CI], 2.13–9.60) and hookah ownership (OR, 10.67; 95% CI, 4.83–23.66) but not with alcohol use (OR, 1.73; 95% CI, 0.74–4.04). Findings also suggest hookah is perceived as a safer alternative to cigarette smoking. Almost 30% of those who never smoked hookah reported they would consider smoking hookah in the future.

**Conclusion:**

Hookah smoking is popular among college students. Misperceptions associated with hookah use indicate a starting point for developing health behavior change interventions. Future studies should investigate social and behavioral determinants of hookah use and determine the incidence of hookah use among college and high school students. Tobacco control activities should include prevention of hookah tobacco use in university settings.

## Introduction

Tobacco use is the single most preventable cause of death in the United States (1). Although the 2014 Surgeon General’s report, *The Health Consequences of Smoking — 50 Years of Progress*, indicates that the prevalence of current cigarette smoking is on the decline, the report emphasizes the need to further monitor patterns of use for all tobacco products, particularly as disparities in use persist and alternate forms of tobacco use are increasing in popularity among youths (1). Hookah tobacco smoking, for example, has increased tremendously (2,3). Hookah, also known as water pipe or shisha, is a device used for smoking tobacco and other substances. Hookah smoking involves passing tobacco smoke through water before inhalation (4). In a typical 1-hour hookah smoking session, hookah users inhale approximately 90,000 mL volume of smoke, which is substantially more smoke than the smoke from 1 cigarette (500–600 mL) (5,6). The charcoal used to heat the tobacco can raise health risks by producing high levels of carbon monoxide, metals, and cancer-causing chemicals (6). One session of hookah use contains approximately 200 puffs of smoke, which exposes users to 3- to 6-fold higher levels of carbon monoxide and 46-fold higher levels of tar than from a single cigarette (6,7).

Globally more than 100 million people use hookah regularly (8). However, given the recent proliferation of hookah cafes worldwide, this estimate is likely to increase (6). In the past decade, 2,000 to 3,000 new hookah cafes opened in the United States alone (9). Until recently, few studies focused on hookah smoking, and this practice was not considered a serious health problem (7,10). Although studies have begun to examine hookah use among college-aged students, the related socio-behavioral risk factors are largely unknown (2,11). Reports do, however, suggest hookah smoking is increasingly popular among youths in the United States (11,12). Thus, the objective of this cross-sectional study was to determine the prevalence of hookah use at a large urban university in south Florida and to describe the knowledge and practices associated with hookah use among university students.

## Methods

This study used a cross-sectional survey design. Subjects were recruited from a convenience sample of students who were attending a large urban university in Florida during the 2011–12 academic year. To be eligible for participation, participants had to be enrolled as a graduate or undergraduate student at the University of South Florida (USF) during the spring 2012 semester. To assess the prevalence of hookah use, a cluster sample of 478 students was recruited. Students were asked to take a self-administered survey that included questions on demographics; current hookah smoking (defined as hookah use in the past 30 days); hookah use during their lifetime; associated risk factors; and knowledge, attitudes, beliefs, and practices regarding hookah use. Questions were designed based on a literature and expert review. Before data collection, all study instruments were field-tested with a sample of 20 students and revised on the basis of the test results.

To determine the required size for the overall sample and to calculate cluster sizes (one-stage cluster sampling), we used the Centers for Disease Control and Prevention’s EpiInfo v.6 Statistical Calculator’s “population study option” (parameters: total population size, expected frequency, and worst acceptable). More than 30 natural clusters were sampled, including all the university’s colleges, 2 libraries, the student center, on-campus dormitories, and the fitness center. The study was reviewed and approved by the university’s institutional review board. All data were analyzed in SAS 9.3 (SAS Institute Inc). First, demographic characteristics of hookah users and nonusers were compared using χ^2^ and Fisher exact tests. Current and lifetime use prevalence was evaluated for the entire sample and subgroups. Finally, unadjusted and adjusted analyses using logistic regression modeling were conducted to identify associated factors for hookah use. Analysis results were adjusted for age, sex, education, cigarette smoking, alcohol use, owning a personal hookah, having a friend who smoked hookah, and proximity to hookah lounges.

## Results

### Sample description

In total, 478 participants were interviewed, of which 261 were women (54.6%) and 217 were men (45.4%) (Table 1), a ratio consistent with the university’s enrollment in 2011–12 (women, 56%; men, 44%; χ^2^ *P* = .540). Most (78.8%) were undergraduate students, a finding also consistent with the USF enrollment (undergraduate, 74%; graduate/postgraduate, 22%; χ^2^ *P* = .781). Participants were of varied ethnicities including white (33.4%), Asian (21.3%), Hispanic (17.4%), and black (12.8%). The sample ethnicity was statistically different (*P* = .001) from the university’s enrollment in 2011–12, which was reported as white (60%), Hispanic (17%), Asian (6%), and black (11%).

**Table 1:** Characteristics of University Students Surveyed About Prevalence, Knowledge, and Practices of Hookah Smoking, Florida, 2012^a^

Characteristics	Total Population (n = 478), n (%)	Hookah Use (n = 76), n (%)	No Hookah Use (n = 389), n (%)	*P* Value^b^
**Sex**				
Female	261 (54.6)	29 (11.5)	224 (88.5)	.002
Male	217 (45.4)	47 (22.2)	165 (77.8)	
**Ethnicity**				
White	154 (33.4)	26 (17.3)	124 (82.7)	.081
Asian	98 (21.3)	16 (16.3)	82 (83.7)	
Hispanic/Latino	80 (17.4)	10 (12.5)	70 (87.5)	
African American/Black	59 (12.8)	2 (3.6)	54 (96.4)	
Middle Eastern	45 (9.8)	10 (24.4)	31 (75.6)	
Native American	5 (1.1)	1 (20.0)	4 (80.0)	
Other	20 (4.2)	5 (26.3)	14 (73.7)	
**Age, y**				
≤20	208 (44.2)	40 (19.6)	164 (80.4)	.429
≥21	263 (55.8)	35 (13.7)	221 (86.3)	
**Study program**				
Undergraduate	372 (78.8)	57 (15.6)	308 (84.4)	.967
Graduate	100 (21.2)	15 (15.8)	80 (84.2)	
**Cigarette use**				
Yes	69 (14.6)	31 (45.6)	37 (54.4)	<.001
No	402 (85.4)	45 (11.3)	352 (88.7)	
**Alcohol use**				
Yes	285 (60.9)	56 (19.9)	225 (80.1)	.007
No	183 (39.1)	19 (10.4)	163 (89.6)	
**Hookah ownership**				
Owns hookah	52 (11.0)	31 (60.8)	20 (39.2)	<.001
Does not own hookah	421 (89.0)	45 (11.0)	366 (89.0)	
**Friends use hookah**				
Yes	367 (85.3)	74 (20.8)	282 (79.2)	<.001
No	63 (14.7)	1 (1.6)	61 (98.4)	

a Missing observations not included in the analyses.

b *P* values <.01 are considered significant.

### Current and lifetime prevalence of hookah smoking

Current prevalence of hookah use was 16.3% (95% CI, 13.0–19.7) (Table 2). More men than women reported current hookah use (22.2% men vs 11.5% women). Students of Middle Eastern descent reported the highest prevalence of current hookah use (24.4%), followed by white (17.3%), Asian (16.3%), Hispanic (12.5%), and African American (3.6%) students. There was no difference in the prevalence of hookah smoking between undergraduate (15.6%) and graduate (15.8%) students.

**Table 2:** Prevalence of Current and Lifetime Hookah Use Among University Students, Florida, 2012

Characteristics	Total Sample, N (%)	Current Hookah Use, % (95% CI)	Lifetime Hookah Use, % (95% CI)
**Overall sample**	478 (100)	16.3 (13.0–19.7)	54.4 (50.0–58.9)
**Sex**			
Female	261 (54.6)	11.5 (7.5–15.4)	49.4 (43.3–55.5)
Male	217 (45.4)	22.2 (16.6–27.8)	60.6 (54.0–67.1)
**Race**			
White	154 (33.4)	17.3 (11.3–23.4)	68.2 (60.8–75.5)
Asian	98 (21.3)	16.3 (9.0–23.6)	42.3 (32.4–52.1)
Hispanic/Latino	80 (17.4)	12.5 (5.2–19.8)	62.0 (51.3–72.7)
African American/black	59 (12.8)	3.6 (0.4–12.3)	28.8 (17.8–42.1)
Middle Eastern	45 (9.8)	24.4 (11.2–37.5)	53.5 (38.6–68.4)
Native American	5 (1.1)	20.0 (0.5–71.6)	20.0 (0.5–71.6)
Others	20 (4.3)	26.3 (6.5–46.1)	55.0 (33.2–76.8)
**Study program**			
Undergraduate	372 (78.8)	15.6 (11.9–19.3)	53.8 (48.7–58.9)
Graduate	100 (21.2)	15.8 (8.5–23.1)	54.1 (44.2–64.0)
**Friends use hookah**			
Yes	367 (85.3)	20.8 (16.6–25.0)	61.1 (56.0–66.1)
No	63 (14.7)	1.6 (0.04–8.7)	39.7 (27.6–51.8)
**Hookah lounge within 10 miles of** **residence**			
Present	379 (92.2)	18.9 (14.9–22.9)	60.4 (55.4–65.3)
Not present	32 (7.8)	13.3 (1.2–25.5)	41.9 (24.6–59.3)
**Hookah ownership**			
Owns hookah	52 (11.0)	60.8 (47.4–74.2)	96.2 (86.8–99.5)
Does not own hookah	421 (89.0)	11.0 (7.9–143.0)	49.5 (44.7–54.3)
**Cigarette use**			
Yes	69 (14.6)	45.6 (33.8–57.4)	91.3 (84.7–98.0)
No	402 (85.4)	11.3 (8.2–14.4)	48.7 (43.8–53.6)
**Alcohol use**			
Yes	285 (60.9)	19.9 (15.3–24.6)	68.1 (62.7–73.5)
No	183 (39.1)	10.4 (6.0–14.9)	34.6 (27.7–41.6)

Abbreviation: CI, confidence interval.

The prevalence of lifetime (ever use) of hookah in our sample was 54.4% (95% CI, 50.0–58.9) with 49.4% of women and 60.6% of men reporting ever using a hookah. Lifetime use by race/ethnicity was 68.2% for white students, 62.0% for Hispanic students, 53.5% for Middle Eastern students, 42.3% for Asian students, 28.8% for African American students, and 20.0% for Native American students. Lifetime use among undergraduates was 53.8% and among graduates was 54.1%. Additionally, prevalence of lifetime use was 2-fold higher among participants who owned a private hookah (96.2%) than among those who did not (49.5%).

### Knowledge, attitudes, and practices of hookah smoking

When shown a picture of a standard hookah, most (95.8%) participants recognized the image. When asked about the harmfulness of hookah smoking, 74.6% indicated that hookah smoking is harmful for health. However, 12.6% reported hookah smoking was not harmful, and 12.8% reported they were unsure of the harmfulness. Most (50.6%) participants also indicated that cigarette smoking is more dangerous than hookah smoking. When asked to identify the sources from which they received information on hookah harmfulness, participants cited no formal means of acquiring data about hookah safety or harm; they instead reported using their own judgment (70.3%) or acquiring information from friends (25.9%) or the Internet (22.4%). When asked about the presence of hookah bars or lounges, 92.2% of participants reported having a hookah bar or lounge within a 10-mile radius of their residential area.

Those who had not smoked hookah were asked if they would ever consider hookah smoking in the future. Almost 30% reported they would. Of those who reported they would consider hookah smoking in the future, reasons cited for this were the time together with friends, the fun associated with the activity, the pleasant atmosphere, the social acceptability, and the perception that hookah smoking was a healthier alterative to cigarette use.

Most (85.3%) respondents reported having a friend who smokes hookah. More than 30% of the sample reported having a friend who owns a hookah, while 11% of respondents reported owning one themselves. Current hookah usage was 6-fold higher among participants who owned a private hookah (60.8%) when compared with those who did not (11.0%). Also, prevalence of current hookah use was 4-fold higher among cigarette smokers (45.6%) than among those who did not smoke cigarettes (11.3%).

Current hookah users were asked how often they smoke hookah in a given month. Most (55.3%) reported smoking hookah once in a month, with fewer smoking hookah twice a week (19.7%), once a week (19.7%), or every day (5.3%). When asked about length of exposure, almost half of current hookah smokers reported smoking hookah for longer than 1 hour per session, with 32.4% smoking for 61 to 120 minutes and 13.5% smoking greater than 120 minutes per session. Another 40.5% of participants reported spending between 30 and 60 minutes a session and 13.5% reported smoking for less than 30 minutes.

### Factors associated with hookah smoking

Unadjusted and adjusted logistic regression modeling was conducted to determine the independent association between socio-demographic factors and hookah use as well as knowledge, attitudes, and practices regarding hookah smoking (Table 3). In unadjusted analysis, sex and race were significantly associated with increased odds of hookah smoking. Men were 2.2 times more likely than women to use hookah (odds ratio [OR], 2.2; 95% confidence interval [CI], 1.33–3.64). When compared with whites, African Americans (OR, 0.18; 95% CI, 0.04–0.77) had significantly lower risk of hookah smoking; the lower risk among Hispanics (OR, 0.68; 95% CI, 0.31–1.50) was insignificant. The increased risk among Middle Easterners (OR, 1.05; 95% CI, 0.56–1.96) was not significant.

**Table 3:** Factors Associated With Hookah Use Among University Students (n = 478), Florida, 2012

Characteristics	OR (95% CI)	AOR (95% CI)
**Age**	0.97 (0.90–1.04)	0.94 (0.81–1.01)
**Sex**		
Male	2.20 (1.33–3.64)	1.60 (0.84–3.08)
Female	1 [Reference]	1 [Reference]
**Race**		
African American/black	0.18 (0.04–0.77)	0.46 (0.08–2.69)
Hispanic/Latino	0.68 (0.31–1.50)	0.75 (0.28–1.97)
Middle Eastern	1.05 (0.56–1.96)	0.97 (0.29–3.26)
Other	1.54 (0.67–3.52)	1.69 (0.75–3.81)
White	1 [Reference]	1 [Reference]
**Study program**		
Undergraduate	0.99 (0.53–1.83)	0.49 (0.17–1.39)
Graduate	1 [Reference]	1 [Reference]
**Cigarette use**		
Yes	6.55 (3.71–11.58)	4.52 (2.13–9.60)
No	1 [Reference]	1 [Reference]
**Alcohol use**		
Yes	2.14 (1.22–3.73)	1.73 (0.74–4.04)
No	1 [Reference]	1 [Reference]
**Hookah ownership**		
Owns hookah	12.61 (6.64–23.95)	10.67 (4.83–23.66)
Does not own hookah	1 [Reference]	1 [Reference]
**Friends use hookah**		
Yes	16.00 (2.18–117.37)	7.44 (0.83–67.01)
No	1 [Reference]	1 [Reference]
**Hookah lounge within 10 miles of** **residence**		
Present	1.52 (0.51–4.49)	1.25 (0.28–5.47)
Not present	1 [Reference]	1 [Reference]

Abbreviations: OR, odds ratio; CI, confidence interval; AOR, adjusted odds ratio.

Cigarette smoking, alcohol use, hookah ownership, and having a friend who uses hookah were other significant risk factors in the unadjusted model. However, in the adjusted model, only cigarette smoking and hookah ownership remained significantly associated with hookah use (controlling for sex, alcohol use, having friends who use hookah, and having a hookah bar or lounge within 10 miles of one’s residence). Specifically, cigarette smokers were 4.52 times more likely than nonsmokers to use hookah. Moreover, hookah ownership increased 10.67-fold the risk of hookah use.

## Discussion

Findings from our study suggest the current prevalence of hookah use among college students at USF is 16.3%. This rate is consistent with prevalence studies conducted with college-aged youths (11,13,14). Findings are also consistent with those of other studies, including higher rates of smoking among men than among women, higher prevalence among non-Hispanic whites than among other races, and higher prevalence among smokers than among nonsmokers (13,14). The prevalence of ever using hookah (54.4%) found in our study was higher than estimates reported by Fielder and colleagues (15) in New York (about 45%) and by Smith and colleagues (14) in California (about 25%).

The value of our study lies in the descriptive nature of the knowledge, attitudes, and practices of the current student population, which shed light for potential preventive actions in university settings. In particular, our study indicates 2 important factors that must be addressed to curtail hookah smoking: the social nature of hookah use and misperceptions regarding risk and harmfulness of hookah smoking reported by nearly a quarter of our sample. Other studies pointed to the importance of these factors as predictors of increased prevalence of hookah smoking during college (11,14). However, specific interventions are needed to dispel each different belief of the population at risk. For instance, most respondents acknowledged that hookah smoking has harmful effects, findings consistent with other studies (10). Yet more than half of the sample believed hookah smoking to be a safer alternative to cigarette smoking and, regardless of their perception of harmfulness, almost a third of the sample reported they would consider hookah smoking in the future. This finding suggests that knowledge regarding the harmful effects of hookah use may not be the most substantial barrier to preventing hookah smoking and that correcting the misperception of hookah as a safer alternative may be a more appropriate target for health education efforts. Further investigations into risk factors for hookah smoking initiation and continuation are needed (14).

The reasons why individuals believe hookah smoking is a safer alternative to cigarette smoking are unclear. However, there is no evidence that the effects of the tobacco are less serious if a water pipe is used (16). In our study, more than half of the sample perceived cigarette smoking as more dangerous than hookah smoking. Yet data suggest that hookah smoking, because of higher levels of carbon monoxide and tar, poses a considerably greater health hazard than cigarette smoking (6,9). Because of the filtration mechanism, smoke that emits from a hookah is softer and lighter and has a more pleasant smell than smoke emitted from a cigarette. This may lead smokers to a false belief that hookah smoking is safe or harmless (17). Traditional antitobacco campaigns focus on cigarettes, cigars, and smokeless tobacco with little to no attention to hookah or other nicotine delivery systems, potentially further spreading this false belief.

One notable reason that students consider hookah smoking is the social nature of the activity, which may be a difficult barrier for health education programs to overcome. Hookah smoking brings with it numerous challenges not combated by previous antismoking campaigns, including the social aspect of relaxing with friends and the fun associated with this activity. The high rate of hookah device ownership in our study suggests that hookah smoking may be moving from an occasional activity to a regular habit. However, we could not find studies conducted in the same setting with which to compare our study findings. In Florida, efforts have been made to track tobacco use (including hookah) among high school students through population-based surveys (18), but data for college students are not gathered. Efforts are beginning at the national level to track tobacco use (including hookah) among college students in the United States, but institutionally based studies are scarce. The absence of local data is a limitation for direct local action. This situation should prompt universities to monitor alternative forms of tobacco use, including hookah, e-cigarettes, and emerging alternatives, in local student health assessments.

Our findings suggest the need for population-based studies to examine unintended effects of current regulations on alternative forms of tobacco use. For instance, although Florida’s Clean Indoor Air Act regulates “any lighted tobacco product,” (19) many hookah lounges are licensed as retail tobacco stores, exempting them from the act. This loophole in the law creates the appearance that hookah smoking may be permitted in places where cigarette and cigar smoking is prohibited, which may be perceived as acknowledgment that hookah smoking is safe for the public. Under current regulations, hookah can be purchased from a hookah lounge or an online shop without age restriction and hookah products are widely available in convenience stores at lower prices than cigarettes, making hookah an available and viable alternative to cigarettes. Additionally, the lack of regulations on the proximity of hookah smoking cafés to university settings is also concerning. Some have recommended regulations to prevent hookah establishments from operating near high schools or colleges, enforcement of strict identity checks, and the taxing of hookah products as potential ways to combat the rising trends in hookah use (12,20). More studies, both qualitative and quantitative, are needed to examine the role of popular media in promoting new hookah establishments near universities to evaluate whether hookah is being portrayed as a “healthy” alternative to smoking.

As with any research initiative, this study is not without limitation. Although numerous instruments for measuring hookah use have been reported (21), we could not find a standardized tool available to estimate the prevalence of hookah use. Thus, our instrument was designed based on available data in the literature. Although this study makes an important step in developing such a tool (ie, our instrument was pre-piloted, piloted, and field tested, and revised multiple times before data collection), assessment of factorial validity and cross-validation in large samples was outside the scope of our study. Future studies should attempt to assess the psychometric properties of available research instruments on larger samples. Another limitation is the cross-sectional nature of our data. Specifically, our findings suggest cigarette smoking is associated with hookah use. However, because of the cross-sectional data, such association should be taken with caution and must be further explored in longitudinal studies that can characterize risk more appropriately. More studies are needed to understand the link between cigarette usage and hookah usage. Future studies should compare the frequency and intensity of usage between hookah and cigarette smoking. For instance, we were not able to assess whether students were substituting cigarette use with hookah use or whether they were using both smoking methods in tandem. Cigarette smokers may indeed constitute a high-risk group for hookah smoking, in which case additional efforts should be aimed at cigarette smokers to increase their awareness of the harmfulness of all alternative smoking mechanisms, including hookah. Conversely, hookah smoking may be a gateway for smoking. Our data do not permit such distinction, and we recommend that panel studies be conducted in university settings to uncover why prevalence changes are occurring. Future studies should attempt to disentangle the relationship between concurrent cigarette smoking and hookah use. Because of the cross-sectional nature of the data and the lack of comparison data from previous years, we could not assess trends. Because current trends in water pipe use in other universities indicate that water pipe use is increasing among college students (11,14,22), future studies must attempt to examine hookah usage longitudinally and by year in university.

Our findings indicate a need to monitor knowledge, attitudes, and practices related to hookah use among college students. Misperceptions associated with hookah use are a starting point for the development of health behavior change interventions. On the basis of our findings, we recommend that public health messaging consider misperceptions regarding hookah use as a safe alternative to cigarettes and target youths and college students as well as the general public. Educational campaigns must be designed to address misunderstandings regarding risks associated with hookah smoking and should be inclusive of other recreational tobacco use and nicotine delivery devices, including e-cigarettes, e-hookah, and other emerging devices, as well as the regulation of sales and marking of these devices. Future studies should attempt to gain a deeper understanding of the social and behavioral determinants of hookah use and determine the incidence of hookah use in representative samples of college and high school students. Tobacco control activities should include prevention of water pipe tobacco use in university settings.
